# Health behaviors and health status of Korean middle-aged men by marital status: Korea Community Health Study, 2015

**DOI:** 10.4178/epih.e2019019

**Published:** 2019-05-15

**Authors:** Yongho Jee, Youngtae Cho

**Affiliations:** Department of Public Health Science, Graduate School of Public Health, Seoul National University, Seoul, Korea

**Keywords:** Marital status, Health behavior, Health status, Middle aged men

## Abstract

**OBJECTIVES:**

Previous studies have shown that marital status is associated with household composition and living arrangements, which partially explain observed differences in health status according to marital status. However, due to the rapid socioeconomic and demographic transformations of the last few decades, the distribution of marital status among middle-aged adults has become more diverse. Therefore, this study aimed to obtain up-to-date information on the associations between marital status and health and to investigate the implications of these findings for conventional explanations of the health effects of marriage.

**METHODS:**

The data for this study were obtained from the 2015 Korean Community Health Study. We compared 4 modifiable lifestyle behaviors—smoking, alcohol consumption, physical activity, and self-rated health status—as outcome variables in association with marital status in Korean middle-aged men (age 40-44) living in Seoul and other regions.

**RESULTS:**

Married men showed the lowest cigarette smoking prevalence and the highest subjective health status both before and after adjusting for education and income. The odds of engaging in vigorous physical activity did not show a major difference before and after adjustment for income and education.

**CONCLUSIONS:**

In married men, the prevalence of cigarette smoking was lowest and subjective health status was highest, similar to previous studies. However, the prevalence of engaging in physical activity was highest in divorced/widowed/separated men. The health behaviors and health status of Korean middle-aged adults should be more closely followed, since they are representative of demographic changes in the Korean population.

## INTRODUCTION

Marital status is regarded as one of the most important social determinants of health. Most previous studies have reported that married people are healthier in terms of mortality and morbidity than their unmarried, never-married, divorced, or widowed counterparts [1-3]. Marriage protection and marriage selection are the major theories explaining the trend for better health in the married population [3]. The marriage protection theory suggests that married people have more advantages in terms of economic resources, social and psychological support, and support for healthy lifestyles (e.g., to avoid cigarette smoking and alcohol/drug abuse). Cohort studies in the USA have reported that becoming divorced or widowed was associated with decreased vegetable intake, increased alcohol consumption [4], and an increased risk of restarting or initiating smoking in women [5]. The marital selection theory instead proposes that healthier people get married and stay married, while less healthy people either do not get married or are more likely to become divorced or widowed. Previous studies have reported that marital status is associated with household composition and living arrangements, which partially explain observed differences in health status according to marital status [6-8].

Over the last few decades, many countries have undergone economic and social transformations, resulting in changes in familial and marital patterns such as increases in the average age at marriage and the number of international marriages, and a decrease in the stigma of divorce [9,10]. It has been observed that changes in social norms regarding marriage were associated with adults remaining single [11].

As a result of rapid economic growth and the 1997-1998 financial crisis, Korea has experienced rapid demographic shifts and labor market flexibility, with a trend for insecure employment to be associated with an increased age of first marriage [12].

In particular, Korean men who are currently in their 40s (born in 1970s) have experienced the rise and fall of the Korean economy, in a sense, since they entered the job market during the period of the 1997-1998 financial crisis. They are the transitional cohort following the “baby boom”, when the total fertility rate was above 3.0. Moreover, those middle-aged men are known to be a vulnerable risk group in terms of various health conditions [13,14].

Therefore, given the unique historical background of this age group in Korea and the growing evidence for associations between marital status and health-related risks, exploring the health status of Korean middle-aged men according to marital status has important implications for examining demographic changes in Korean society. The main aim of this study was to investigate the health behaviors and health status of Korean middle-aged men according to marital status. If more evidence regarding this issue is reported in further follow-up studies, future governmental campaigns should refect modified social norms on marriage and childbearing.

## MATERIALS AND METHODS

### Subjects

**Figure 1.**
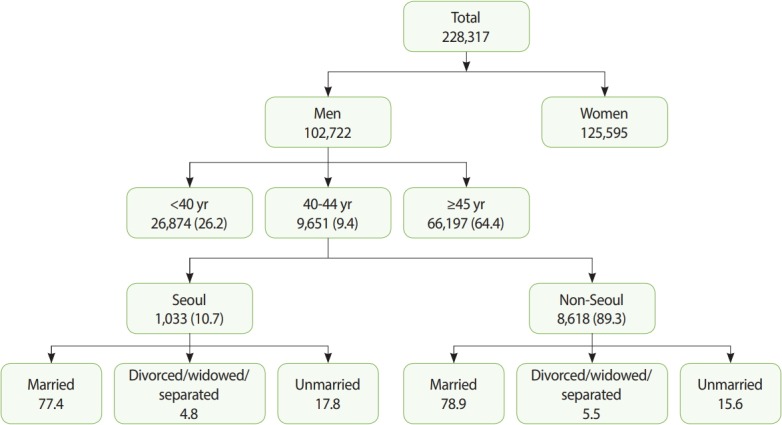
Participants of our study: the 2015 Korea Community Health Survey dataset. Values are presented as number of participant and %.

The data for this study were obtained from the Korean Community Health Study (KCHS) of 2015. The KCHS, which is conducted by the Korea Centers for Disease Control and Prevention, is the only community-based study existing in Korea. The KCHS is an annual, nationally representative, cross-sectional survey conducted at 253 regional sites in Korea, and its results are made available for public use. The study was launched in 2008 to provide data for population-based estimates of health indicators to support the evidence-based development and assessment of public health policies. Using multi-stage sampling, the KCHS was designed to recruit a representative sample of adults aged 19 years and older. The 2015 KCHS database contained pooled data from 228,317 interviewees (102,722 men, 125,595 women). Of these, 9,651 men who were aged 40-44 years and lived in Seoul and other regions were included in our final analysis (Figure 1).

### Study variables

All variables contained in the KCHS were measured based on self-reported information. In the selection of lifestyle variables to analyze in the study, we referred to previous studies of the relationships between marital status and risky health behaviors [2,3,5,15,16]. We examined 4 modifiable lifestyle behaviors: smoking, alcohol consumption, physical activity (PA), and self-rated health status. The subjects were divided into 6 groups according to geographical region (Seoul, other regions) and marital status (married, divorced/bereaved/separated, and unmarried). The geographical dichotomization of our participants was chosen in light of the socioeconomic gap between the Seoul metropolitan area and other regions, with support from the fact that previous studies have reported health gaps between urban and rural regions [17-19].

Smoking status was categorized into 3 groups: current smoking, former smoking, and non-smoking. The category of current smoking was defined as including people who had smoked more than 100 cigarettes in their lifetime among those who reported smoking daily or occasionally. Alcohol consumption was defined as including people who drank alcohol more than once a month during the last year. PA was categorized into vigorous PA, moderate PA, and walking. Vigorous PA was defined as engaging in any kind of PA that increased one’s heart rate and caused one to breathe hard for 3 or more days per week for 20 minutes or more per day. Moderate PA was defined as any kind of PA that increased one’s heart rate slightly for 3 or more days per week for 20 minutes or more per day. Participants were considered to engage in walking if they did so for 5 or more days per week for 30 minutes or more per day.

### Statistical analysis

The estimated prevalence of the 4 health behaviors according to marital status and region were obtained using the PROC SURVEYFREQ procedure. The PROC SURVEYLOGISTIC procedure was used to perform multivariate logistic regression analysis to estimate the odds ratios (ORs) and 95% confidence intervals (CIs) of the associations of health behaviors with socio-demographic factors. In the multivariable analysis, for each of the health behaviors, married men were used as the reference group. All statistical analyses were conducted using SAS version 9.4 (SAS Institute Inc., Cary, NC, USA).

### Ethics statement

Data from the KCHS survey are made publicly available through the KCHS website (https://chs.cdc.go.kr/chs/index.do). Thus, ethical approval was not required for this study.

## RESULTS

We divided our results into crude results and multivariable results. Among the 9,651 men aged 40-44 years, 1,033 lived in Seoul (10.7%) and 8,618 lived in other regions (89.3%). Among the 1,033 men who lived in Seoul, 799 (77.4%) were married, 50 were divorced/widowed/separated (4.8%), and 184 (17.8%) were unmarried. Among the 8,618 men who lived outside of Seoul, 7,695 (78.9%) were married, 474 were divorced/widowed/separated (5.5%), and 1,344 (15.6%) were unmarried (Figure 1).

Current smoking was least common among married men in all regions. Statistically significant differences in smoking prevalence were observed among the men aged 40-44 years. However, the proportion of alcohol drinkers was highest among men who were married. The prevalence of alcohol drinking showed statistically significant differences in overall participants and in those from regions other than Seoul. In terms of PA, divorced/widowed/separated men showed the highest prevalence of engaging in vigorous PA, and unmarried men had the highest prevalence of engaging in walking, while married men had the lowest prevalence of engaging in moderate PA and walking. Subjective health status was highest among married men, both in Seoul and in other regions (Table 1). In a crude model, the odds of being a current smoker were 2.74 times higher among divorced/widowed/separated men than among married men (OR, 2.74; 95% CI, 2.07 to 3.62; model 1 of current smokers) (Table 2). The association remained statistically significant even after adjusting for education level and household income level (OR, 2.46; 95% CI, 1.84 to 3.30). However, no major difference was found in the OR for former smoking before or after adjustment (Table 2). For alcohol consumption, in contrast to the results of the crude analysis, which showed that married men had the highest prevalence of alcohol drinking, the statistical significance of that difference weakened after adjusting for income and education level (Table 2). The odds of engaging in vigorous PA did not show a major difference before and after adjustment for income and education (Table 3). Subjective health status was highest among married men before and after adjusting for income and education (Table 3).

## DISCUSSION

Most chronic diseases are associated with multiple unhealthy lifestyle factors, including smoking, unhealthy diet, sedentary activity, and heavy alcohol consumption [20].

The long-term health effects from multiple unhealthy lifestyle patterns tend to show interactions, rather than simply adding to each other [21,22]; therefore, lifestyle modification has been considered as an effective intervention strategy for the management of various chronic diseases. Our study aimed to investigate the health status and health behavior of Korean middle-aged men according to marital status in order to compare our results with those of previous studies regarding familial or marital status and health behaviors. The majority of previous studies reported that married adults and parents tended to be healthier than their unmarried and nonparent counterparts in terms of age-adjusted mortality and various health behaviors [23,24]. The protective effects of marital and parental status on mortality have usually been explained by the positive effects of social integration or social support; the familial relationships of marriage and parenting may provide external regulation and facilitate self-regulation of health behaviors [24].

A study conducted by Durkheim [25] highlighted the importance of the parent-child relationship as a source of social integration preventing suicide, as suicide rates were found to be higher for the childless than for parents. Gove stressed that the state of being married is associated with (1) psychological well-being, leading to a reduced risk of certain cause of death (e.g., accidents, homicide, and suicide); (2) being less likely to engage in activities leading to death (e.g., smoking and alcohol drinking); and (3) more motivation and capability to undergo treatment for diseases [26]. Our data partially support the findings of previous studies, although some of our results are inconsistent with previous reports. Married men showed the lowest prevalence of cigarette smoking and the highest scores for subjective health status, as in previous studies [18,19]. However, the prevalence of engaging in PA was highest in divorced/widowed/separated men. Since widowed men are likely to be considerably older, on average, they may be more likely to engage in PA for practical reasons, in response to physical signals regarding their health. However, the high prevalence of alcohol drinking among married men was an unexpected result that does not fit with the conventional theories of marital status and health.

In Korea, the proportion of single-person households has rapidly increased throughout the last 15 years, and is predicted to reach 35% in 2035 [20]. The primary reasons for this phenomenon are the low birth rate and the growing number of late marriages. Additionally, the proportion of unmarried and single middle-aged adults is steadily increasing, in terms of both the proportion and the absolute number, with roughly 1 in 5 householders living alone in 2015 [20]. However, not all single-person households are necessarily non-divorced or divorced individuals; they could reflect couples who maintain separate households due to work circumstances, or so called ‘goose fathers’ (fathers who work and live alone, while their wives and children reside abroad). However, since the proportion of single-person households is increasing across various age groups, it is necessary to carefully examine the health status of unmarried and single-person households in order to effectively manage their health.

The present study has some limitations due to its cross-sectional design; thus, the results require cautious interpretation in terms of residual confounding and reverse causation bias. Nevertheless, future research should aim to establish temporality and to enhance our understanding of the association between marital status and health conditions by comparing different generations of adults. The lack of consideration of job-related variables such as occupation type, labor hours, and employment stability is another limitation regarding the sample selection.

In our study, married men showed the lowest prevalence of cigarette smoking and the highest subjective health status, as in previous studies. However, in contrast to previous studies, the prevalence of engaging in PA was highest in divorced/widowed/separated men. The health behaviors and health status of Korean middle-aged adults should be more closely followed since they are representative of demographic changes in the Korean population.

## Figures and Tables

**Table 1.** Health status of participants by region and marital status (%)

Variables	Seoul				Other regions			
	Married	Divorced, widowed, or separated	Unmarried	p-value (χ^2^-test)	Married	Divorced, widowed, or separated	Unmarried	p-value (χ^2^-test)
Smoking status								
Non-smoking	22.9	12.0	26.6		20.9	11.3	20.4	
Ex-smoking	33.7	28.0	24.5	0.002	30.1	15.8	20.4	<0.001
Current smoking	43.4	60.0	48.9		49.0	72.9	59.3	
Alcohol consumption								
Non-drinking	15.9	12.0	23.4	0.032	17.8	19.1	25.2	<0.001
Drinking	84.1	88.0	76.6		82.2	80.9	74.8	
Physical activity (yes)								
Vigorous	19.7	30.0	23.9		21.0	21.0	17.2	
Moderate	8.9	16.0	13.0	0.118	13.3	15.3	14.2	0.005
Walking	54.6	66.0	61.4		33.1	35.1	39.2	
Subjective health status								
Excellent/very good	48.8	48.0	39.7	0.081	48.4	44.8	43.0	<0.001
Others	51.2	52.0	60.3		51.6	55.3	57.0	

**Table 2.** Multivariate analysis with smoking and drinking as dependent variables

Variables	Smoking				Drinking	
	Model 1^1^	Model 2^1^	Model 3^2^	Model 4^2^	Model 1	Model 2
Marital status						
Married	1.00 (reference)	1.00 (reference)	1.00 (reference)	1.00 (reference)	1.00 (reference)	1.00 (reference)
Divorced, widowed, or separated	2.74 (2.07, 3.62)	2.46 (1.84, 3.30)	1.03 (0.74, 1.43)	0.99 (0.70, 1.40)	0.95 (0.75, 1.19)	1.18 (0.93, 1.49)
Unmarried	1.20 (1.04, 1.38)	1.06 (0.91, 1.24)	0.68 (0.58, 0.81)	0.69 (0.57, 0.82)	0.64 (0.56, 0.73)	0.84 (0.73, 0.97)
Education level (high)		0.64 (0.60, 0.67)		0.83 (0.78, 0.88)		0.91 (0.87, 0.96)
Household income level (high)		1.06 (1.02, 1.10)		1.06 (1.02, 1.10)		1.21 (1.17, 1.26)
Region						
Seoul		1.00 (reference)		1.00 (reference)		1.00 (reference)
Other regions		1.08 (0.91, 1.28)		0.93 (0.77, 1.12)		0.93 (0.78, 1.11)

Values are presents as odds ratio (95% confidence interval).

1 Current smoking as a dependent variable.

2 Former smoking as a dependent variable.

**Table 3.** Multivariate analysis with no vigorous physical activity, smoking, and subjective health status as dependent variables

Variables	Vigorous physical activity (no)		Walking (no)		Subjective health status (other than excellent or very good)	
	Model 1	Model 2	Model 1	Model 2	Model 1	Model 2
Marital status						
Married	1.00 (reference)	1.00 (reference)	1.00 (reference)	1.00 (reference)	1.00 (reference)	1.00 (reference)
Divorced, widowed or separated	1.06 (0.85, 1.31)	1.13 (0.90, 1.41)	1.12 (0.93, 1.34)	1.12 (0.92, 1.35)	1.15 (0.96, 1.37)	1.04 (0.86, 1.24)
Unmarried	0.83 (0.72, 0.96)	0.90 (0.77, 1.05)	1.32 (1.18, 1.47)	1.30 (1.15, 1.46)	1.27 (1.14, 1.42)	1.13 (1.01, 1.28)
Education level (high)		1.02 (0.97, 1.07)		1.04 (0.99, 1.08)		0.90 (0.86, 0.93)
Household income level (high)		1.05 (1.02, 1.09)		0.98 (0.96, 1.01)		0.96 (0.93, 0.99)
Region						
Seoul		1.00 (reference)		1.00 (reference)		1.00 (reference)
Other regions		1.02 (0.86, 1.20)		0.41 (0.36, 0.47)		0.94 (0.82, 1.07)

Values are presented as odds ratio (95% confidence interval).
